# Prevalence of FMS-like tyrosine kinase 3/internal tandem duplication (FLT3/ITD+) in de novo acute myeloid leukemia patients categorized according to cytogenetic risk

**DOI:** 10.1590/S1516-31802009000100006

**Published:** 2009-05-11

**Authors:** Everson Augusto Krum, Mihoko Yamamoto, Maria de Lourdes Lopes Ferrari Chauffaille

**Affiliations:** 1 PhD. Professor, Universidade Estadual de Ponta Grossa (UEPG), Ponta Grossa, Paraná, Brazil.; 2 MD, PhD. Associate professor, Universidade Federal de São Paulo - Escola Paulista de Medicina (Unifesp-EPM), São Paulo, Brazil.

**Keywords:** Receptor protein - tyrosine kinase, Leukemia, myeloid, acute, Cytogenetic analysis, Prognosis, Polymerase chain reaction, Receptores proteína tirosina quinases, Leucemia mielóide aguda, Análise citogenética, Prognóstico, Reação em cadeia da polimerase

## Abstract

**CONTEXT AND OBJECTIVE:**

The mechanism involved in leukemogenesis remains unclear and more information about the disruption of the cell proliferation, cell differentiation and apoptosis of neoplastic cells is required.

**DESIGN AND SETTING:**

Cross-sectional prevalence study at the Discipline of Hematology, Hospital São Paulo, Universidade Federal de São Paulo.

**METHODS:**

We investigated FMS-like tyrosine kinase 3/internal tandem duplication (FLT3/ITD+) in 40 adult patients with de novo acute myeloid leukemia (AML), categorized according to cytogenetic results, from September 2001 to May 2005.

**RESULTS:**

Thirteen patients (32.5%) were classified as presenting the favorable karyotype, 11 patients (27.5%) as an intermediate group, 7 patients (17%) as an undefined group and 9 patients (22.5%) as the unfavorable group. FLT3/ITD^+^ was found in 10 patients (25%): 3 with FLT3/ITD^+^ and favorable karyotype; 4 with FLT3/ITD^+^ and intermediate karyotype; 2 with FLT3/ITD^+^ and undefined karyotype; and only 1 with FLT3/ITD^+^ and unfavorable karyotype. Among the patients without FLT3/ITD^+^, 10 presented favorable karyotype, 8 intermediate, 4 undefined and 8 unfavorable karyotype. The cytogenetic results showed no correlations between FLT3/ITD presence and the prognostic groups (P = 0.13). We found that 2 patients were still alive more than 24 months later, FLT3/ITD^+^ did not influence the patients’ survival rate.

**CONCLUSION:**

We found the same frequency of AML with FLT3/ITD^+^ in both the favorable and intermediate prognosis groups. Only one patient presented AML, FLT3/ITD^+^ and unfavorable karyotype (the hypothetical worst clinical situation). Therefore, the prognostic advantage of favorable cytogenetics among patients with FLT3/ITD^+^ remains to be elucidated, for it to be better understood.

## RESUMO

**CONTEXTO E OBJETIVO:**

O mecanismo envolvido na leucemogênese permanece obscuro, e maiores informações a respeito das inadequadas proliferação, diferenciação e apoptose das células neoplásicas é fundamental.

**TIPO DE ESTUDO E LOCAL:**

Estudo transversal de prevalência na Disciplina de Hematologia e Hemoterapia, Hospital São Paulo, Universidade Federal de São Paulo.

**MÉTODOS:**

Nós pesquisamos a duplicação interna in tandem (DIT) do gene FLT3 (Fms-like tyrosine kinase) em 40 pacientes adultos com leucemia mielóide aguda (LMA) de novo, classificados de acordo com os resultados de cariótipo em banda G, de setembro de2001 a maio de 2005.

**RESULTADOS:**

Treze pacientes (32,5%) foram classificados como cariótipo favorável, 11 pacientes (27,5%) como grupo intermediário, 7 pacientes (17%) no grupo de prognóstico indefinido e os restantes 9 pacientes (22,5%) foram alocados como desfavorável. A DIT/FLT3 foi encontrada em 10 pacientes (25%), 3 pacientes com DIT/FLT3 e cariótipo favorável, 4 com DIT/FLT3 e cariótipo intermediário, 2 com DIT/FLT3 e cariótipo de prognóstico indefinido e somente 1 paciente com DIT/FLT3 e cariótipo desfavorável. Entre os pacientes sem DIT/FLT3, 10 apresentaram cariótipo favorável, 8 com cariótipo intermediário, 4 com cariótipo de prognóstico indefinido e 8 com cariótipo desfavorável. Não houve correlação entre a presença de DIT/FLT3 e os grupos de prognóstico conforme resultados de citogenética (P = 0,13). No presente estudo encontramos 2 pacientes vivos por mais de 24 meses. A presença de DIT/FLT3 não influenciou a taxa de sobrevida dos pacientes.

**CONCLUSÃO:**

Nós observamos a mesma frequência de LMA com DIT/FLT3 tanto no grupo de cariótipo favorável quanto no grupo intermediário e somente um paciente com LMA e DIT/FLT3 e cariótipo desfavorável, hipoteticamente o pior achado clínico. Desta forma, a vantagem prognóstica do cariótipo favorável em pacientes DIT/FLT3 permanece a ser esclarecida para melhor compreensão da LMA.

## INTRODUCTION

Acute myeloid leukemia (AML) is a clonal disorder that involves mutated hematopoietic precursors with inappropriate properties (e.g. uncontrolled proliferation, absence of apoptosis, abnormal cell differentiation, etc.).^1^ The crucial question is which parts are defective in the multistep anomaly-generating process of genetic programming of AML cells. According to the “two hit” model of leukemogenesis,^2^ class I mutations confer a proliferative and/or survival advantage to cells, while class II mutations give rise to impaired differentiation and abnormal apoptosis. Despite the important role of FMS-like tyrosine kinase 3/internal tandem duplication (FLT3/ITD+), a class I mutation, in providing a myeloproliferative signal and/or survival advantage to leukemia cells, this mutation does not affect differentiation by itself. Thus, for AML onset, additional class II mutations such as chromosome rearrangements are probably required,^3^ e.g. promyelocytic leukemia-retinoic acid receptor-alpha (PML-RARa), core binding factor/smooth muscle myosin heavy chain (CBFß/SMMHC), mixed-lineage leukemia (MLL) and acute myeloid leukemia-1 transcription factor/eight-twenty-one corepressor (AML1/ETO). These result in abnormalities relating to the CBF complex, MLL, Hox proteins and retinoic acid receptors.^1^

The FLT3 gene encodes an important tyrosine kinase receptor that is critical for normal hematopoiesis.^4^ FLT3/ITD^+^^5^ promotes disruption of a repressor sequence in the juxtamembrane domain of the FLT3 gene, leading to constitutive autophosphorylation of the receptor,^6^ thereby promoting proliferation and inhibiting apoptosis in leukemic cells.^6^^,^^7^ FLT3/ITD^+^ is found in 20% to 30% of de novo AML patients^7^^,^^8^^,^^9^^,^^10^ and is the most mutated gene in leukemia, with a significant clinical impact on patients.^11^

We approached this subject by investigating FLT3/ITD^+^ in de novo AML patients categorized according to cytogenetic results, focusing especially on the group with favorable prognosis and normal karyotype, and associating this with age and white blood cell (WBC) count.

## METHODS

### Patient samples

The eligible cases consisted of adult patients referred to Hospital São Paulo, Universidade Federal de São Paulo - Escola Paulista de Medicina (Unifesp-EPM) from September 2001 to May 2005 (Table 1), with de novo AML according to World Health Organization diagnostic criteria.^12^ Immunophenotypic cell characterization was performed using a FACScalibur flow cytometer (Becton Dickinson, California, United States) with standardized monoclonal antibodies, in accordance with previously published protocols. The Unifesp Ethics Committee approved this study.

**Table 1.** Cytogenetic findings, World Health Organization (WHO) criteria, prognostic group stratification and presence of FMS-like tyrosine kinase 3/internal tandem duplication (FLT3/ITD)

Patient	age/sex	WHO criteria	Karyotype	Prognostic group stratification	FLT3/ITD
1	21/M	A	46,XY,t(15;17)(q22;q11)	Favorable	ITD -
2	71/F	D	46,XX,add(21)(q22)	Intermediate	ITD -
3	50/F	C	53-56,XX, +1,+8, +9,+10,i?(11)(q13), +14,+15, +16,+17,+18,+19, 6dmin[cp20]	Unfavorable	ITD -
4	70/M	B	46,XY	Intermediate	ITD+
5	70/F	D	Unavailable	Undefined	ITD -
6	73/F	D	Unavailable	Undefined	ITD -
7	62/M	B	46,XY,+14,+22/46,XY	Unfavorable	ITD -
8	56/M	D	46,XY,del(3)(q31)/46,XY	Unfavorable	ITD -
9	41/F	D	Unavailable	Undefined	ITD -
10	41/F	A	46.XX,t(15;17)(q22;q12)	Favorable	ITD+
11	22/M	A	46,XY,t(15;17)(q22;q11)	Favorable	ITD+
12	81/M	D	46,XY	Intermediate	ITD -
13	80/F	D	46,XX	Intermediate	ITD+
14	63/F	A	46,XX,t(8;21)(q22;q22)	Favorable	ITD -
15	43/M	B	46,XY	Intermediate	ITD -
16	46/M	D	46,XY,t(1;2)(p31;q34)	Intermediate	ITD -
17	29/M	D	Unavailable	Undefined	ITD -
18	38/M	A	46,XY,t(15;17)(q22;q21)	Favorable	ITD -
19	91/F	B	48,XX,+8+8	Unfavorable	ITD -
20	52/F	A	46,XX,del(11)(q23)/46,XX	Unfavorable	ITD -
21	22/M	A	46,XY,t(8;21)(q22;q22)	Favorable	ITD+
22	63/F	D	46,XX,t(1;10)(q44;q43)/46,XX	Intermediate	ITD -
23	24/F	A	47,XX,+9,inv(16)(p13;q22)	Favorable	ITD -
24	29/F	A	46,XX,+8,inv(16)/47,XX,inv(16),+22	Favorable	ITD -
25	75/F	D	86~167, XXXX<7n>/46,XX	Unfavorable	ITD+
26	38/F	A	46,XX,inv(16)(p13;q22)/46,XX	Favorable	ITD -
27	63/M	A	46,XX,t(8;21)(q22;q22)	Favorable	ITD -
28	87/F	B	47,XX,+8/46,XX	Intermediate	ITD+
29	29/M	D	46,XY,t(9;11)(p22;q11.2)	Intermediate	ITD -
30	67/M	D	Unavailable	Undefined	ITD+
31	54/M	D	45,X,-Y,del(5)(q15;q33),del(7)(p13)	Unfavorable	ITD -
32	35/F	D	Unavailable	Undefined	ITD+
33	52/F	A	46,XX,t(2;13)t(8;21)(q22;q22)	Favorable	ITD -
34	73/F	B	46,XX,der(19),t(1;19)(q21;p13)/46,XX	Intermediate	ITD -
35	30/M	A	46,XY,del(11)(q23)	Unfavorable	ITD -
36	32/M	D	Unavailable	Undefined	ITD+
37	41/F	D	47,XX,t(3;5)(q25;q34),+4	Unfavorable	ITD -
38	78/F	D	46,XX	Intermediate	ITD -
39	48/M	A	46,XY,t(8;21)(q22;q22)	Favorable	ITD -
40	54/F	A	46,XX,t(8;21)(q22;q22)	Favorable	ITD -

A = acute myeloid leukemia with recurrent chromosomal abnormalities; B = acute myeloid leukemia with multilineage dysplasia; C = acute myeloid leukemia after therapy (chemo or radio); D = acute myeloid leukemia not catego rized; M = male; F = female.

### Cytogenetics

Chromosome analyses were performed on short-term non-stimulated cultures from bone marrow cells. These were harvested after 24 hours and slides from these cultures were banded using the Trypsin-Giemsa technique (GTG).^13^ The metaphases were analyzed using an image capture system (Olympus BX microscope and Cytovision data processing through Applied Biosystems). The abnormalities were described in accordance with the International System for Human Cytogenetic Nomenclature (ISCN).^14^ The karyotype results were categorized as the following prognostic groups: favorable, with t(8;21), t(15;17) or inv(16); intermediate, with normal karyotype or structural or numerical changes not encompassed by the favorable or adverse risk groups; undefined, with metaphases unavailable; and unfavorable, with complex karyotype, monosomy of chromosome 5 or 7, deletions of the long arm of chromosome 5 or 3q abnormalities.^15^

### Polymerase chain reaction (PCR)

Deoxyribonucleic acid (DNA) from bone marrow cells was obtained by means of proteinase K digestion, phenol-chloroform extraction and ethanol precipitation. This was then analyzed for the presence of FLT3/ITD^+^ by means of the polymerase chain reaction using published primers (ITD/Dialab) for exons 14 and 15.^16^ The amplification products were analyzed on 2% agarose gel and stained with ethidium bromide.

## RESULTS

Forty eligible patients were enrolled: 18 men and 22 women (male/female ratio of 0.82). The median age was 52 years (range 21-91 years). Karyotyping was successful in the cases of 33 patients (83%), while seven samples were unavailable (17%). According to the karyotyping results, the patients were classified into prognostic groups, stratified as shown in Table 1.

Thirteen patients (32.5%) were classified as presenting a karyotype with a favorable prognosis: six with t(8;21) (cases 14, 21, 27, 33, 39 and 40); four with t(15;17) (cases 1, 10, 11 and 18) and three with inv(16) (cases 23, 24 and 26). Eleven patients (27.5%) were placed in the intermediate karyotype group: five with normal karyotype (cases 4, 12, 13, 15 and 38); two with abnormalities of chromosome 1 (cases 16 and 22); one with isolated trisomy 8 (case 28); one with translocation of chromosome 9 (case 29); one with translocation of chromosome 19 (case 34); and one with abnormal chromosome 21 (case 2). Seven patients (17%) were placed in the undefined karyotype group (cases 5, 6, 9, 17, 30, 32 and 36). The remaining nine patients (22.5%) were categorized in the unfavorable group: two patients with complex karyotype (cases 3 and 25); two with abnormalities of chromosome 3 (cases 8 and 37); one with del(5q) and del(7p) (case 31); one with tetrasomy 8 (case 19); two with 11q23 abnormalities (cases 20 and 35) and one with trisomy 22 and +14 (case 7).

FLT3/ITD^+^ was found in 10 patients (25%): three with FLT3/ITD^+^ and favorable karyotype; four with FLT3/ITD^+^ and intermediate karyotype; two with FLT3/ITD^+^ and undefined karyotype; and only one patient with FLT3/ITD^+^ and unfavorable karyotype. There was no difference in prognosis stratification group regarding presence or absence of FLT3/ITD^+^ (P = 0.13). Among the patients without FLT3/ITD^+^, 10 presented favorable karyotype, eight intermediate, four undefined and eight unfavorable karyotype.

The median age of the patients with favorable karyotype was 38 years, while it was 54 years in the unfavorable group. Patients with FLT3/ITD^+^ had a median age of 54 years (range 22-87 years), while patients without FLT3/ITD^+^ had a median age of 53 years (range 21-91 years) (P = 0.97).

Patients with FLT3/ITD^+^ had a median white blood cell (WBC) count of 9.2 x 10^9^/l (range: 0.6 x 10^9^/l to 202 x 10^9^/l) and patients without FLT3/ITD^+^ had a median WBC count of 12.1 x 10^9^/l (range: 1.9 x 10^9^/l to 240 x 10^9^/l) (P = 0.22). Two patients with FLT3/ITD^+^ had a WBC count of up to 50 x 10^9^/l, while seven patients without FLT3/ITD^+^ had a WBC count of up to 50 x 10^9^/l. The median WBC count of the patients with unfavorable karyotype was 21 x 10^9^/l, while it was 9.7 x 10^9^/l in the intermediate group and 9.3 x 10^9^/l in the group with favorable karyotype. Patients with FLT3/ITD^+^ had a mean WBC count of 30.4 x 10^9^/l, while patients without FLT3/ITD^+^ had a mean WBC count of 45.3 x 10^9^/l.

## DISCUSSION

The real mechanism involved in the transformation from normal to leukemic cells remains unclear, and therefore some further light on this issue is needed. The presence of an abnormal karyotype in association with FLT3/ITD^+^ might be one factor implicated in leukemogenesis, although not the only one. There is much evidence that chromosomal abnormalities and alterations to genomic DNA are implicated in deregulated proliferation, escape of apoptosis or defective apoptosis and subsequent excessive survival of neoplastic cells.

In the present study, we found that 25% of the patients presented FLT3/ITD^+^. According to the chromosomal abnormality classification, 13 patients had a favorable karyotype: three (23%) with FLT3/ITD^+^ and 10 without mutations. Other studies have reported that patients with FLT3/ITD^+^ had a poor prognosis, but in our study, we found that cases 11 and 21 remained alive for 31 and 27 months respectively, while only one patient (case 10) had a short survival (one month) after diagnosis. In fact, FLT3/ITD^+^ did not influence the patients’ survival rate. The favorable chromosome rearrangement may have prevailed over the FLT3 gene mutation.

Considering the importance and frequency of recurrent chromosomal abnormalities, AML with translocation t(8;21) results in an AML1/ETO fusion gene involved in blocking AML-1 dependent transcription activation. It also blocks the activating effects of CCAAT/enhancer-binding protein-alpha (C/EBPa), which is responsible for deregulated cell differentiation.^17^^,^^18^ We found AML with t(8;21) in 14% of our patients and in one with FLT3/ITD^+^ (2.5%). This demonstrates that fusion rearrangement is not fundamental but contributes towards other genetic alterations such as mutations like FLT3/ITD^+^,^19^ and towards leukemic events in some situations that are not fully understood. In fact, we recently reported on one case of myelodysplastic syndrome (MDS) in a follow-up study. This patient underwent a transformation to leukemia with AML/ETO fusion and acquisition of FLT3/ITD^+^, thereby corroborating Gilliland’s “two hit” hypothesis for leukemogenesis.^20^

The CBF complex acts on genes that are crucial for myeloid proliferation. According to Reilly,^21^ potential leukemic cells have CBF fusion genes but another hit is required for AML development. In cases of inversion of chromosome 16, the fusion protein CBFß/SMMHC is produced, which is a CBF inhibitor resulting in abnormal transcription of proliferative signals.^6^ In AML cases with inversion of chromosome 16, the consequence is inactivation of the CBFß protein, abnormal transcription of the proliferative signal and blocking of cell maturation.^6^^,^^17^ We observed AML with inv(16) in 7.5% of our patients and none with FLT3/ITD^+^.

In leukemia with t(15;17), blocking of cell differentiation is a consequence from the PML/RAR-a fusion protein, which leads to abnormal functioning of the retinoic acid receptor alpha.^22^ AML with t(15;17) was found in four of our patients (10% of the cases), and two of them had FLT3/ITD^+^. This feature corroborates other studies that found that about half of the patients had PML/RAR-a but did not have FLT3/ITD^+^.^9^^,^^10^^,^^11^^,^^19^ With regard to patients with AML and normal karyotype, we observed that 12.5% of our patients had AML and normal karyotype, thus differing from other studies that found that 45-50% had normal karyotype.^15^^,^^23^^,^^24^ The incidence of FLT3/ITD^+^ and normal karyotype in the present study was 40%, while Whitman et al.^25^ reported this in 28% of their patients. Among the seven patients in our study without metaphases available, we found that three had FLT3/ITD^+^ (42.8%), thus showing the need to apply several methods in order to comprehensively identify the disease, using other resources such as the PCR technique to characterize AML patients.^26^

In our study, the data are conflicting. We did not find any statistical difference in mean WBC counts between FLT3/ITD^+^ patients and patients without FLT3/ITD^+^ (P = 0.224). The median WBC count of patients with an unfavorable karyotype was higher than in cases with an intermediate or favorable karyotype. This shows that the higher count for the unfavorable group is a further factor for a poor prognosis. There was no age difference between AML patients with FLT3/ITD^+^ and those without FLT3/ITD^+^, in agreement with a recent report by Levis and Small.^11^ Altogether, for patients with de novo AML, the best approach includes cytogenetic analysis and subsequent categorization into favorable, intermediate, undefined or unfavorable groups and investigation of FLT3/ITD^+^.^24^

## CONCLUSION

In conclusion, we found the same frequency of AML with FLT3/ITD^+^ in both the favorable and intermediate prognosis groups (3/10 and 4/10 patients were positive, respectively). There was only one patient with AML, FLT3/ITD^+^ and unfavorable karyotype (i.e. the hypothetical worst clinical situation). The prognostic advantage of favorable cytogenetics among patients with FLT3/ITD^+^ remains to be elucidated and the clinical significance is a matter of controversy. Thus, further investigation is warranted.
